# Supplementary material for: Genetic effects on variability in visual aesthetic evaluations are partially shared across visual domains
Source: Commun Biol. 2024 Jan 6;7:55. doi: 10.1038/s42003-023-05710-4 (PMC10771521; doi:10.1038/s42003-023-05710-4)
Supplement: Supplementary file 2 — Supplementary Information [file 42003_2023_5710_MOESM2_ESM.pdf]

Supplementary Information for *Genetic effects on variability in visual aesthetic evaluations are partially shared across visual domains*

Table of Contents

SUPPLEMENTARY NOTES 2

SUPPLEMENTARY NOTE 1: INTRA-RATER TEST-RETEST RELIABILITY AND EXCLUSION CRITERIA 2
SUPPLEMENTARY NOTE 2: INTRA-IMAGE TEST-RETEST RELIABILITY 3
SUPPLEMENTARY NOTE 3: MULTILEVEL MODELLING AND VARIANCE PARTITIONING COEFFICIENTS 4
SUPPLEMENTARY NOTE 4: TASTE-TYPICALLY COMPARISON WITH PAIRWISE AESTHETIC AGREEMENT 5
SUPPLEMENTARY NOTE 5: PRINCIPAL COMPONENT ANALYSIS 6
SUPPLEMENTARY NOTE 6: MONOZYGOTIC TWINS SHOW HIGHER PAIRWISE AESTHETIC AGREEMENT THAN DIZYGOTIC TWINS AND UNRELATED PAIRS: RESULTS WITH OUTLIERS AND FROM THE VALIDATION SAMPLE 7
SUPPLEMENTARY NOTE 7: WITHIN-DAY TEST-RETEST RELIABILITIES FOR TASTE-TYPICALITY AND EVALUATION-BIAS 8
SUPPLEMENTARY NOTE 8: BETWEEN-DAY RELIABILITIES FOR PAIRWISE AESTHETIC AGREEMENT, TASTE-TYPICALITY, AND EVALUATION-BIAS 9
SUPPLEMENTARY NOTE 9: PHENOTYPIC CORRELATIONS REPLICATE IN A THIRD NON-OVERLAPPING SAMPLE 10
SUPPLEMENTARY NOTE 10: POWER ANALYSIS 11
SUPPLEMENTARY NOTE 11: SAMPLE OVERLAP 12

SUPPLEMENTARY FIGURES 13

SUPPLEMENTARY FIGURE 1. *INTRA-RATER RELIABILITY THRESHOLD.* 13
SUPPLEMENTARY FIGURE 2. *INTRA-IMAGE RELIABILITY.* 14
SUPPLEMENTARY FIGURE 3. *VARIANCE COMPONENT ANALYSIS FOR AESTHETIC RATINGS.* 15
SUPPLEMENTARY FIGURE 4. *SCATTER PLOT OF THE RELATIONSHIP BETWEEN PAIRWISE AGREEMENT AND TASTE-TYPICALITY.* 16
SUPPLEMENTARY FIGURE 5. *PCA OF INDIVIDUAL AESTHETIC RATINGS.* 17
SUPPLEMENTARY FIGURE 6. *PAIRWISE AGREEMENT DISTRIBUTION AND ANALYSIS IN THE MAIN SAMPLE* [1]. 18
SUPPLEMENTARY FIGURE 7. PAIRWISE AGREEMENT DISTRIBUTION AND ANALYSIS IN THE VALIDATION SAMPLE [2]. 19
SUPPLEMENTARY FIGURE 8. *PHENOTYPIC CORRELATIONS PERSIST AFTER SENSITIVITY ANALYSIS.* 20
SUPPLEMENTARY FIGURE 9. *BIVARIATE HERITABILITY AND GENETIC AND ENVIRONMENTAL CORRELATIONS PERSIST AFTER SENSITIVITY ANALYSIS.* 21
SUPPLEMENTARY FIGURE 10. *POWER ANALYSIS.* 22
SUPPLEMENTARY FIGURE 11. *POWER ANALYSIS IN THE VALIDATION SAMPLE.* 23
SUPPLEMENTARY FIGURE 12. SAMPLE OVERLAP. 24

SUPPLEMENTARY TABLES 25

SUPPLEMENTARY TABLE 1: *PHENOTYPIC TWIN CORRELATIONS BEFORE AND AFTER CONTROLLING FOR CONFOUNDING EFFECTS* 25
SUPPLEMENTARY TABLE 2: *UNIVARIATE MODELLING OF GENETIC AND ENVIRONMENTAL CONTRIBUTIONS TO INTER-INDIVIDUAL DIFFERENCES IN AESTHETIC EVALUATION AFTER CONTROLLING FOR CONFOUNDING EFFECTS* 26

SUPPLEMENTARY REFERENCES 27

Supplementary Notes

Supplementary Note 1: Intra-rater test-retest reliability and exclusion criteria

Similarly to Germine et al. [1] and Sutherland et al. [2], we excluded participants with ratings *sd* = 0, within each visual domain, indicating no variation in ratings. Additionally, similarly to Vessel et al. and Chen et al., we excluded participants $R_{XX\text{-}intra}$ < .5 within each visual domain, indicating poor test-retest reliability in ratings (see Supplementary Figure 1).

$R_{XX\text{-}intra}$ was computed within each participant as the Pearson correlation of their rating for the repeated images per visual domain. $R_{XX\text{-}intra}$ computed means and *sd* were equal to .85 (*sd* = .50) for abstract images, .94 (*sd* = .61) for scenes, and .78 (*sd* = .28) for faces in the original sample, and .92 (*sd* = .46) for scenes and .80 (*sd* = .25) for faces in the validation sample. Thus, the resulting $R_{XX\text{-}intra}$ estimates were similar across samples.

Supplementary Note 2: Intra-image test-retest reliability

We calculated image reliability by computing $R_{XX\text{-}image}$ as the Pearson correlation between each repeated image within each visual domain. To avoid familial effects, we computed reliabilities only on one twin per pair. We detect no $R_{XX\text{-}image} < .5$ across visual domains, samples and twins (Supplementary Figure 2). This indicates that images evoked sufficiently reliable responses across visual domains and suggested that no further exclusion was needed.

Supplementary Note 3: Multilevel modelling and variance partitioning coefficients

Supplementary Figure 3 shows the Variance Partitioning Coefficients (VPC) for the ratings for abstract images, scenes, and faces in the Germine et al. and the Sutherland et al. samples.

VPC were stable across both twins and samples, meaning that participant evaluations across samples were consistent (see Supplementary Figure 3a). The models showed that a substantial part of the variance was accounted for by image and individual-level characteristics and their interaction. Notably, the image term accounted for the greatest variance for both scenes and faces. In contrast, the image by individual interaction term captured the most variance for abstract images. This indicates that the variance shared across the subjects gradually turns into shared as the visual images evaluated gained significance, from abstract to scenes and faces. These results are consistent with previous findings [3,5,6], and theoretical predictions [3,7].

An interesting finding is that the exposure by individual and the image by exposure interactions VCPs explained some, albeit little, of the variance in aesthetic evaluations of images of faces (1% of the total variance, both in the first and the validation sample, and across pair members) and abstract images (1% of the total variance; across pair members). On the one hand, some faces were systematically prone to elicit mere exposure effects (i.e., they led to systematic changes in aesthetic ratings after only one repetition), while other faces did not. On the other hand, this means that some participants were more susceptible to mere exposure effects than others, but apparently only for abstract images. These effects were small and thus did not impact the repeated ratings averaging procedure applied in the main analysis. However, the finding that some of the variance was explained by the block interaction components, even only after one exposure, is a notable finding that deserves further investigation.

To aid the interpretability and comparability of the results, we also computed the beholder Index type 2 (bi_2 [8]), which quantifies in a simple metric idiosyncratic versus shared taste. bi_2 was modified to accommodate the introduction of the random effect of repeated images (block). Type 2 was chosen over type 1 due to our specific hypothesis on the meaningfulness of the subject term [8]. The modified beholder Index type 2 (mBi) was calculated to estimate repeatable variance (variance that was not due to residual variance) due to idiosyncratic responses. The shared repeatable variance was calculated as 1 – mBi (Supplementary Figure 3b). Further details on VPC and, more generally, Variance Component Analysis (VCA) can be found at https://github.com/giacomobignardi/empirical-aesthetics-VCA/tree/main/01_VCA.

Supplementary Note 4: Taste-typically comparison with pairwise aesthetic agreement

We quantified the relationship between taste-typicality and pairwise aesthetic agreement (r_{inter}) by correlating the r_{inter} values of pairs with the absolute distance of the *mm2* scores of the members of such pairs ($\Delta mm2_{ij} = |mm2_j - mm2_i|$, with *i* and j being member of one pair). The Δ*mm2* predicted, on average, only a small percentage of variance in their pairwise agreement for abstract images, $r^2 = .07$, and images of scenes, $R^2 = .12$, and faces, $R^2 = .14$. Results were similar in the validation sample (scenes, $R^2 = .10$, and faces, $R^2 = .14$). This indicates that taste-typicality is only partially informative regarding the similarity of aesthetic preferences within pairs (see Supplementary Figure 4). We also note that taste-typicality and pairwise agreement, in principle, are mainly dissociated constructs. To explain this further, we imagine an extreme situation in which one participant *i* has a rating vector for 10 images equal to v_i = [5,5,5,5,5,1,1,1,1,1], while another participant *j* has a rating vector equal to v_j = [1,1,1,1,1,5,5,5,5,5]. Assume that the mean rating vector for every other participant is $v_{(n-i-j)}$ = [1,5,5,5,1,1,5,5,5,1], with *n* equal to the entire sample of participants. Then $r_{inter(ij)} = r(v_i,v_j) = -1$; $mm2_i = r(v_i,v_{(n-i-j)}) = 0$; and $mm2_j = r(v_j,v_{(n-i-j)}) = 0$; That is, in the instance of aesthetic ratings, while two participants can show two identical taste-typicality scores ($\Delta mm2_{ij} = 0$), they can at the same time be in perfect disagreement on what they like ($r_{b(ij)} = -1$). In other words, two members of a pair can be equally distant from the group's average preferences yet be maximally distant from each other preferences.

Supplementary Note 5: Principal component analysis

We conducted Principal Component Analysis (PCA) to reduce the dimensionality of individual ratings, where ratings per image are dimensions and points are individuals. The Kaiser–Meyer–Olkin MSA (Measure of Sampling Adequacy) was MSA = 0.95, MSA = 0.94, and MSA = 0.98, for abstract images, scenes and faces, respectively (first member twin only; MSA = 0.95, MSA = 0.95, and MSA = 0.98, for the other twin members). The scree plot representing the proportion of overall variance explained by each component is represented in Supplementary Figure 5a. Comparable to the results obtained on the first members of a twin pair, the individual scores extracted from the first and the second PC for second members only jointly explained 45%, 43%, and 47% of the individual variance in ratings for abstract images, images of scenes, and images of faces, respectively. Results were similar in the validation sample, with 37% and 50%, and 35%, and 51% of the total variance in individual ratings per scene and faces being jointly explained by the first two PCs. Supplementary Figure 5b maps the facets of aesthetic value, namely evaluation-bias and taste-typicality, to the PC scores. Finally, as shown in Supplementary Figure 5c, PCA indicated our results obtained only with the first members per pair to be robust on the other members. Evaluation-bias scores and taste-typicality were also related to the two major axes of variability in aesthetic values. Pearson correlations between the individual evaluation-bias scores extracted from the first PC were all $r > .99$. Correlations between the second twin members scores extracted from the second component and taste-typicality Fisher z transformed values were $r(709) = 0.75$, 95% CI [0.72, 0.78], for abstract images, $r(760) = 0.73$, 95% CI [0.69, 0.76], for scenes, and $r(752) = 0.66$, 95% CI [0.62, 0.7], for faces (all $p < .001$). Results were replicated in the validation sample, with correlations between the first PC and evaluation-bias scores being all $r > .99$, and correlations between the second PC and taste-typicality equal to $r(620) = 0.71$, 95% CI [0.67, 0.75], for scenes, and $r(602) = 0.58$, 95% CI [0.52, 0.63] for faces (all $p < .001$). These results are consistent with what we reported in the main manuscript. We note that these results cast light on the major dimensions of individual differences in aesthetic value. Recently, Chen et al. [4] showed that visual and auditory taste-typicality scores strongly relate to the first principal component obtained from the standardised aesthetic ratings for such stimuli. Here, we propose that when taking unstandardised ratings, evaluation-bias scores, but not taste-typicality scores, capture the majority of individual variability in aesthetic ratings, with taste-typicality being strongly related to only the second dimension.

Supplementary Note 6: Monozygotic twins show higher pairwise aesthetic agreement than dizygotic twins and unrelated pairs: results with outliers and from the validation sample

To create a population reference for the pairwise aesthetic agreement, we additionally computationally created unrelated pairs (UR) with pseudo-random pairing by matching every second member of a pair with the first member from another pair. UR pairs were matched for sex and did not differ in age within each domain (all Welch two-sample t-tests p > .9). Before analysis, seven pairs were removed due to extreme r_{inter} values (3 UR and 4 MZ pairs). Beyond the main effect of pair class reported in the main manuscript, the type III 3X3 (domain X pair class) ANOVA carried out on Fisher z transformed r_{inter} values (z_{inter}), excluding outliers, revealed a significant large main effect of visual domain, $F(2, 4242) = 1176.6$, $p < .001$ ($\eta_p^2 = 0.36$; 95% CI [0.34, 0.38]). The differences between marginal pairwise agreement averaged across pair classes aligned with multilevel modelling results, with abstract images evoking the highest degree of individual differences, and thus the lower level of pairwise agreement, $r_{inter} = 0.38$ (95% CI [0.36, 0.39]). In contrast, images of scenes and faces evoked higher agreement in aesthetic preferences, $r_{inter} = 0.74$ (95% CI [0.73, 0.75]), $r_{inter} = 0.61$ (95% CI [0.59, 0.62]); all $p < .001$, Bonferroni corrected, Supplementary Figure 6).

Results obtained from the same ANOVA, but without excluding outliers, revealed similar results, with a main effect of visual domain, $F(2, 4263) = 1127.18$, $p < .001$ ($\eta_p^2 = 0.35$; 95% CI [0.32, 0.37]), as well as the small effect of pair class on pairwise aesthetic agreement reported in the main manuscript, $F(2, 4263) = 98.79$, $p < .001$ ($\eta_p^2 = 0.04$; 95% CI [0.03, 0.06]).

Results were replicated in the validation sample (Supplementary Figure 7). After removing six pairs that represented extreme outliers in their pairwise agreement (3 Pseudo-Random Unrelated [UR], 2DZ, and 1MZ), a 2X3 (domain X pair class) ANOVA carried out on the $inter_z$ scores was significant for domain and pair class, and comparable to the main analysis, with a significant main effects of visual domain, $F(1, 1836) = 356.06$, $p < .001$ ($\eta_p^2 = 0.16$; 95% CI [0.13, 0.19]) and pair class, $F(2, 1836) = 38.74$, $p < .001$ ($\eta_p^2 = 0.04$; 95% CI [0.02, 0.06]).

Results were unchanged with the inclusion of outliers, $F(1, 1848) = 340.81$, $p < .001$ ($\eta_p^2 = 0.16$; 95% CI [0.13, 0.19]) and $F(1, 1848) = 340.81$, $p < .001$ ($\eta_p^2 = 0.04$; 95% CI [0.02, 0.05]), respectively. Comparisons of z_{inter} marginal means averaged across domains pair classes were all significant (p< .001) and consistent with the directionality of the effects reported in the original sample. Finally, planned comparisons across pair classes within domains confirmed that MZ> DZ> UR within all domains, all $p < .05$.

Supplementary Note 7: Within-day test-retest reliabilities for taste-typicality and evaluation-bias

Since some degree of unknown unreliability of taste-typicality and evaluation-bias can upwardly bias, and hence confound, estimates of unique environmental effects, we quantified reliabilities (R_{XX}) of such metrics. First, we computed taste-typicality and evaluation-bias separately and only for the repeated images (15 for abstract images, 15 for scenes, and 60 for faces). In the discovery sample, Pearson correlations for the taste-typicality and evaluation-bias within individuals, computed on only one pair per twin, were r = 0.83, 95% CI [0.8, 0.85], r = 0.8, 95% CI [0.77, 0.82], and r = 0.78, 95% CI [0.75, 0.8] for taste-typicality, and r = 0.92, 95% CI [0.91, 0.93], r = 0.93, 95% CI [0.92, 0.94], and r = 0.97, 95% CI [0.97, 0.97] for evaluation bias, for abstract images, scenes, and faces, respectively. These results were similar to the validation sample, with Pearson correlations of r = 0.77, 95% CI [0.74, 0.8] and r = 0.84, 95% CI [0.81, 0.86], and r = 0.93, 95% CI [0.92, 0.94] and r = 0.96, 95% CI [0.95, 0.96] for evaluation-bias (scenes and faces, respectively).

To get a more precise and relevant estimate of the reliability of the metrics computed on the averages of the repeated metrics, as we have done in our main analyses, we also used Intra Class Coefficient (*ICC*) of taste-typicality and evaluation-bias to estimate R_{xx}. In the discovery sample, taste-typicality R_{xx} were all good; *ICC(2,k)* =.90, *ICC(2,k)* =.89, and *ICC(2,k)* =. 87, while evaluation bias R_{xx} were all excellent, *ICC(2,k)* = 0.95, *ICC(2,k)* = 0.96, and *ICC(2,k)* = 0.98. Results were nearly identical in the validation sample: with *ICC(2,k)* =.87 and *ICC(2,k)* =.91 for taste-typicality, and *ICC(2,k)* = 0.96 and *ICC(2,k)* = 0.98, evaluation-bias (scenes and faces, respectively). These results indicate good reliability of taste-typicality and evaluation-bias.

Supplementary Note 8: Between-day reliabilities for pairwise aesthetic agreement, taste-typicality, and evaluation-bias

As an additional sensitivity analysis, since memory effects can inflate reliabilities computed on repeated images, we further quantified the stability of taste typicality and evaluation-bias (following [2]). We analysed a third openly available sample for which repeated ratings of images were obtained across days [2]. We used data from n = 78 unrelated individuals with a mean age of 34 y (sd = 7 y, ranging from 21 to 49 y; 47 women, 30 men, 1 other) available at https://osf.io/35zf8/?view_only=e76c6755dcea4be2adc5b075cae896e8. These individuals followed the same experimental procedure undertaken by the twins in the validation sample, rating 150 images of faces (including 50 repeats) and 74 images of scenes (including 24 repeats), with the addition of taking part in the experiment a second time, after a 3 days delay (More details are given in [2]). This allowed us to compute conservative R_{xx} for individual taste-typicality and evaluation-bias scores and test its stability over time, further complementing the taste-typicality and evaluation-bias reliability analysis with an estimate of pairwise R_{xx} for aesthetic agreement.

On the one end, after removing 3 individuals with $R_{xx\text{-intra}} < .5$ (only for faces, as for scenes there were no individuals with $R_{xx\text{-intra}}$ <. 5), Pearson correlations were *r* = 0.8, 95% CI [0.7, 0.87], and *r* = 0.83, 95% CI [0.74, 0.89], for taste-typicality and *r* = 0.83, 95% CI [0.75, 0.89], and *r* = 0.91, 95% CI [0.86, 0.94] for evaluation-bias, for scenes and faces, respectively. On the other, correlations for pairwise aesthetic agreement computed on random pairs were equal to *r* = 0.72, 95% CI [0.52, 0.84], and *r* = 0.68, 95% CI [0.46, 0.83], for scenes and faces, respectively. For comparison, Intra Class Coefficients (ICC(2,1)) computed within pairs and individuals between scores across days were ICC(2,1) = .80, ICC(2,1) = .83, and good to excellent reliability for taste-typicality ICC(2,1) = .81, ICC(2,1) = .91, and evaluation-bias for scenes and faces, respectively.

Overall, these results show good reliability between days and relatively good stability of the metrics used to assess variability in aesthetic value and indicate little room for unreliable measurement to confound E estimates. Moreover, we also note that results indicate a small decay in repeatability in pairwise aesthetic agreement compared to taste-typicality and evaluation bias. Taste-typicality correlations between days were higher than pair-wise correlations between days, with the difference amounting to Δr = 0.08 and Δr = 0.14 for scenes and faces, respectively. However, these differences were not significant (Fisher's *z* = 0.98, *p* = 0.33 and Fisher's *z* = 1.61, *p* = 0.11). As such, more research using larger samples, more time points, and larger time differences between time points are needed to test whether taste-typicality is more stable than aesthetic agreement over longer periods.

Supplementary Note 9: Phenotypic correlations replicate in a third non-overlapping sample

We replicated phenotypic correlations in the same independent sample used to compute the stability of taste-typicality and evaluation-bias. We followed the same procedure outlined for all samples and computed Pearson correlations between taste-typicality for scenes and faces. Estimates were in line with what we have reported for the other two samples (as reported in main Figure 4), with r = 0.3, 95% CI [0.08, 0.5], p = 0.008, computed from data obtained on the first test, and r = 0.27, 95% CI [0.04, 0.47], p = 0.02, computed on data obtained on the following test (3 days apart, see above). Similar conclusions were reached by computing correlations between evaluation-bias for scenes and faces, with with r = 0.35, 95% CI [0.14, 0.54], p = 0.002, and r = 0.44, 95% CI [0.24, 0.61], p < .001, first and second test, respectively. Thus, we replicated the finding obtained from the other two partially overlapping samples that taste-typicality and evaluation-bias scores tend to be partially shared across visual domains.

Supplementary Note 10: Power analysis

We conducted power analysis to estimate the statistical power to detect shared environmental components (C) for each trait, visual domain, and sample for which an ACE model was expected. Precisely, we generated power curves across different expected C, given the estimated heritability (h^2) and highlighted the power to detect the Cs as estimated in this study.

To conduct analysis, we simulated twin data with the proportion of MZ and DZ present in the samples. For each trait, visual domain, and sample, we fixed the A component based on the estimated h^2. We then computed power analysis based on the χ^2 obtained from the LRT between the full ACE model and the reduced AE models applied to the simulated data. We relied on the observed Weighted non-centrality parameters (Wncp), reflecting the average family-wise contribution to the χ^2 :

$$\text{Wncp } = \frac{\chi^2 \, (df=1)}{N}$$

Where N is equal to the total number of families [10]. To generate power curves, we progressively multiplied the Wncp by an incremental sequence ranging from 1 to 5000. Final power curves across traits, visual domains, and samples (Supplementary Figures 10 and 11, discovery and validation sample, respectively) show the statistical power to detect C, given the observed h^2, across different generated C (ranging from .01 to .35). To contextualise power curves within this study, we highlighted the power curve for the estimated C (e.g., C = .29, taste-typicality for abstract images, discovery sample, see Supplementary Figure 10a).

Although we were reasonably powered to detect C ~.30, the two samples were underpowered to detect smaller C. Furthermore, we highlight that to detect hypothetical true C values ranging from .01 to .07, which correspond to the C estimated in this study for every other metric and domain but taste-typicality for abstract images, a research team would require much more than 5000 pairs of individuals.

Supplementary Note 11: Sample Overlap

To estimate the overlap between the Germine et al. and the Sutherland et al. samples, both drawn from the Australian Twin Registry[11], we randomly drew two samples of 1547 and 1243 individuals from a pool of 40000 simulated individuals. We set 40000 as a conservative estimate from the total number of twins registered in the Australian Twin Registry[12]. We calculated the overlap as the number of individuals present in both. We simulated 10000 random drawn and averaged the sample overlap to obtain the average overlap. We found the average overlap equal to ~48 individuals (*sd* = 7; Supplementary Figure 12).

Supplementary Figures

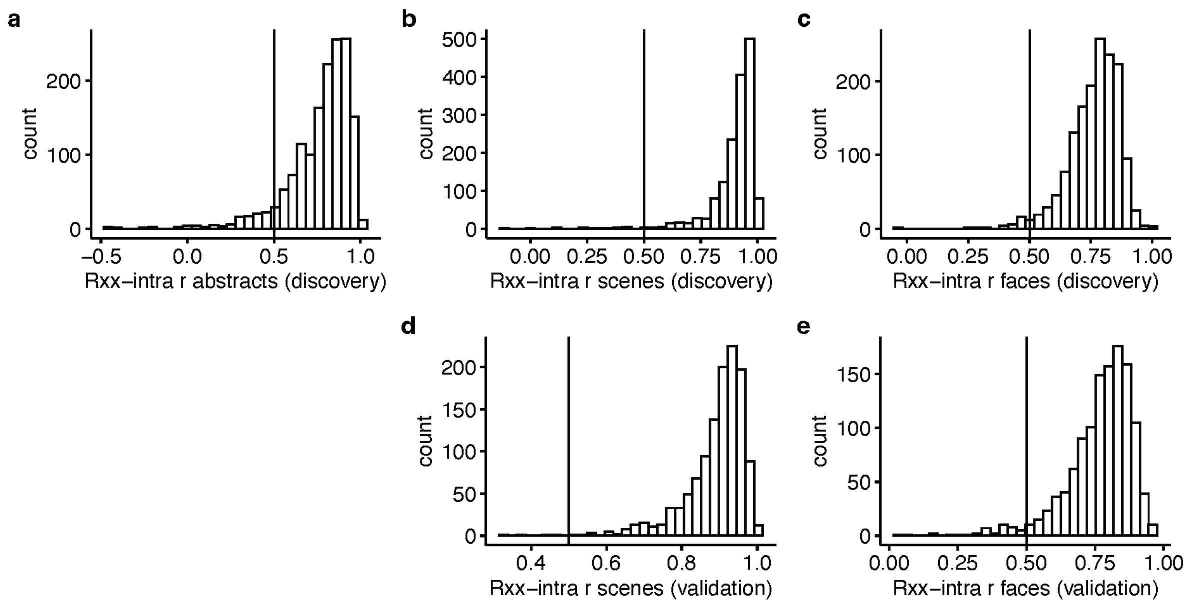

Supplementary Figure 1. *Intra-rater reliability threshold.*

Similarly to Vessel et al.[3] and Chen et al.[4], we excluded participants with $R_{XX\text{-intra}}$ < .5, here represented by the vertical line. Exclusion criteria resulted in the removal of 119, 18, and 32 individuals for further analysis in the discovery sample and 4 and 43 in the validation sample, respectively. Panels **a** to **c** show $R_{XX\text{-intra}}$ histograms from abstract images to faces for the discovery sample. Panels **d** to **e** show $R_{XX\text{-intra}}$ histograms from scenes to faces for the validation sample.

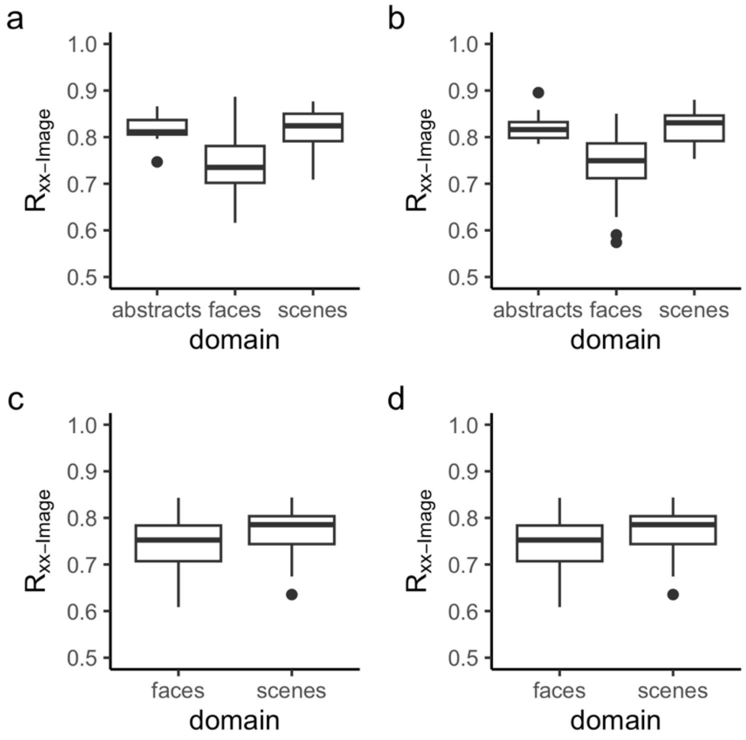

Supplementary Figure 2**.** *Intra-image reliability.*

a-d Box plots of the intra-image reliability ($R_{XX\text{-image}}$) across visual domains for the discovery (**a-b**) and validation samples (**b-d**) for one twin per pair only (left and right, respectively). The horizontal line shows the median; the lower and upper hinges correspond to the 25th and 75th percentiles; the whiskers extend not beyond 1.5*IQR (Inter Quartile Range).

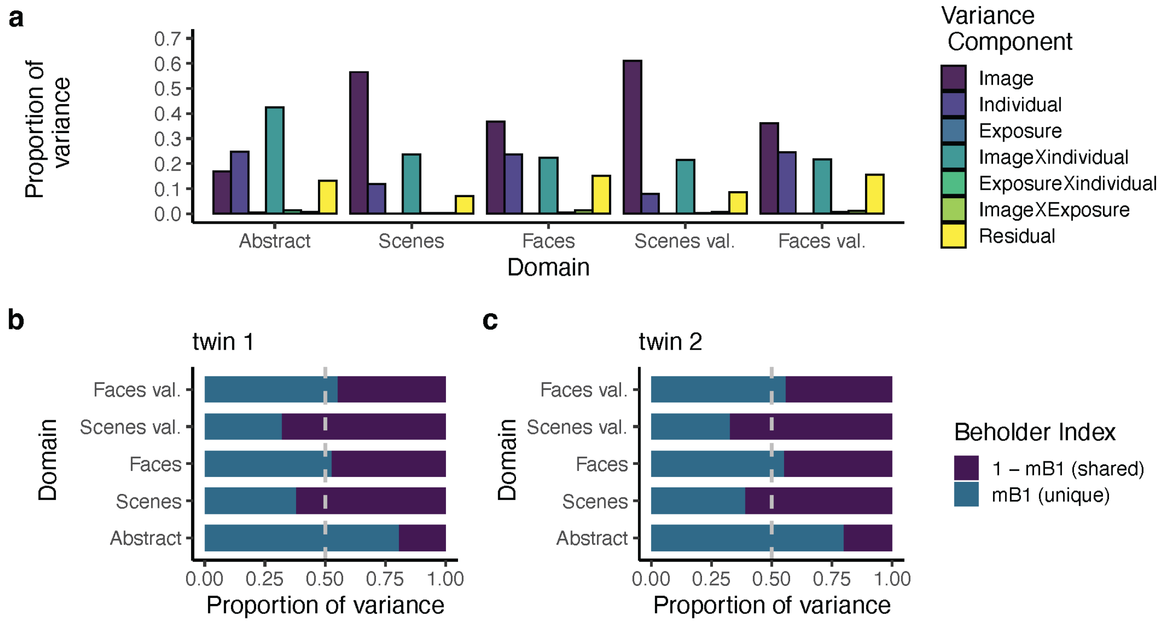

Supplementary Figure 3. *Variance component analysis for aesthetic ratings.*
Panel **a** shows the variance components for aesthetic ratings of abstract images and images of scenes and faces from Germine et al. [1], and images of scenes and faces from the validation sample [2], obtained on the second twin pair members. Panels **b** and **c** show the modified type 2 Hönekopp Beholder index (mBi) [8], which quantifies the amount of variance in aesthetic ratings shared between and unique to individuals for twin 1 and 2, respectively.

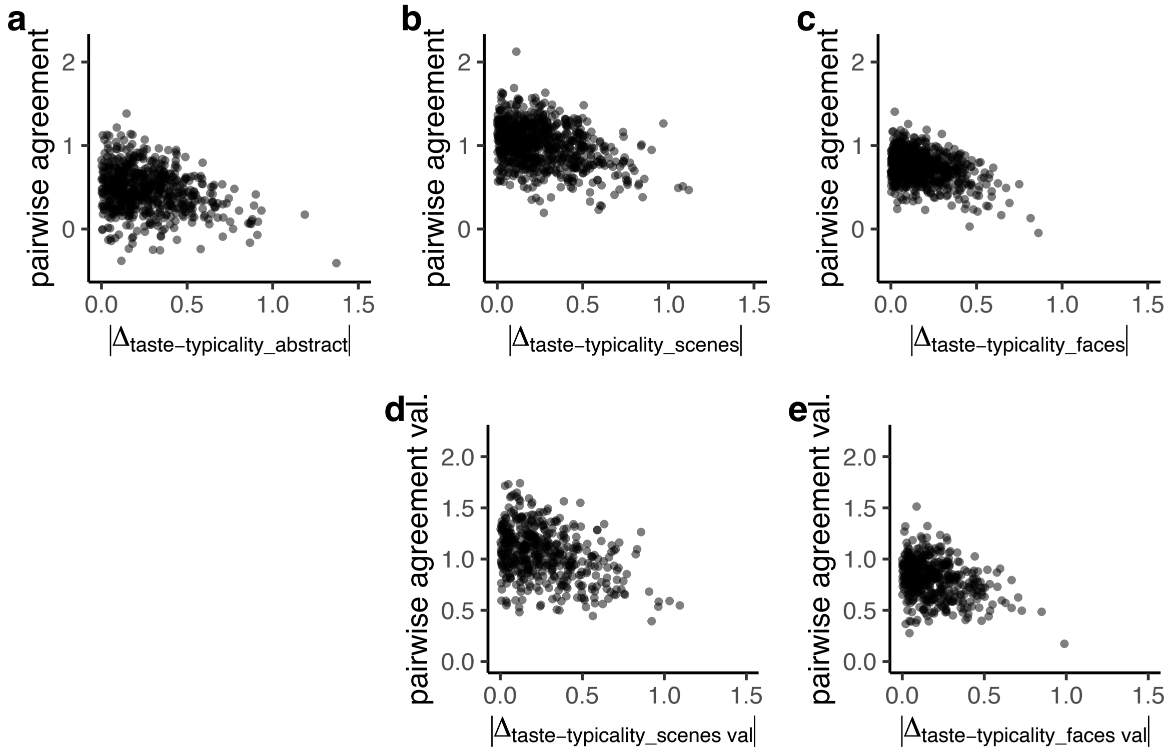

Supplementary Figure 4. *Scatter plot of the relationship between pairwise agreement and taste-typicality.*

Each dot represents the relationship between the pairwise agreement for one pair and their *mm2* similarity score ($|\Delta_{mm2}|$). $|\Delta_{mm2}|$ scores represent the distance between two $mm2_z$ scores within one pair ($\Delta_{mm2} = |mm2_{twin2z} - mm2_{twin1z}|$). Panels **a** to **c** show the scatter plot obtained for the Germine et al. sample; Panels **d** and **e** show the scatter plot for the validation sample

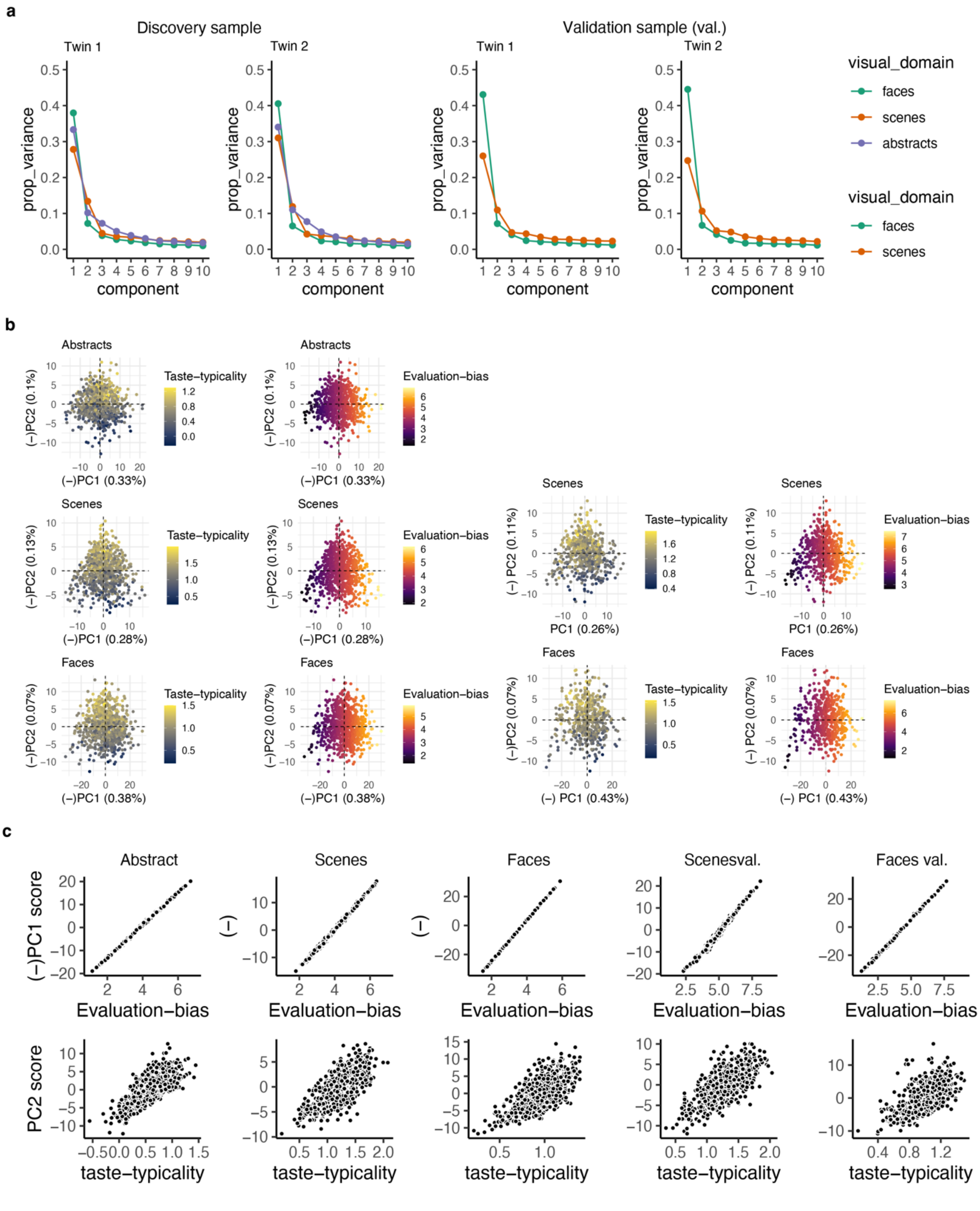

Supplementary Figure 5. *PCA of individual aesthetic ratings*.

Panel **a** shows the scree plot for the PCA over the raw rating data matrix, computed separately per twin membership and samples; Colours represent different visual domains. In Panel **b** taste-typicality and evaluation-bias scores are mapped over the first two component scores. Panel **c** shows the scatter plot and represents the relationship between evaluation-bias and PC1 scores, and between taste-typicality and PC2 scores. Each dot in panels b and c represents one individual.

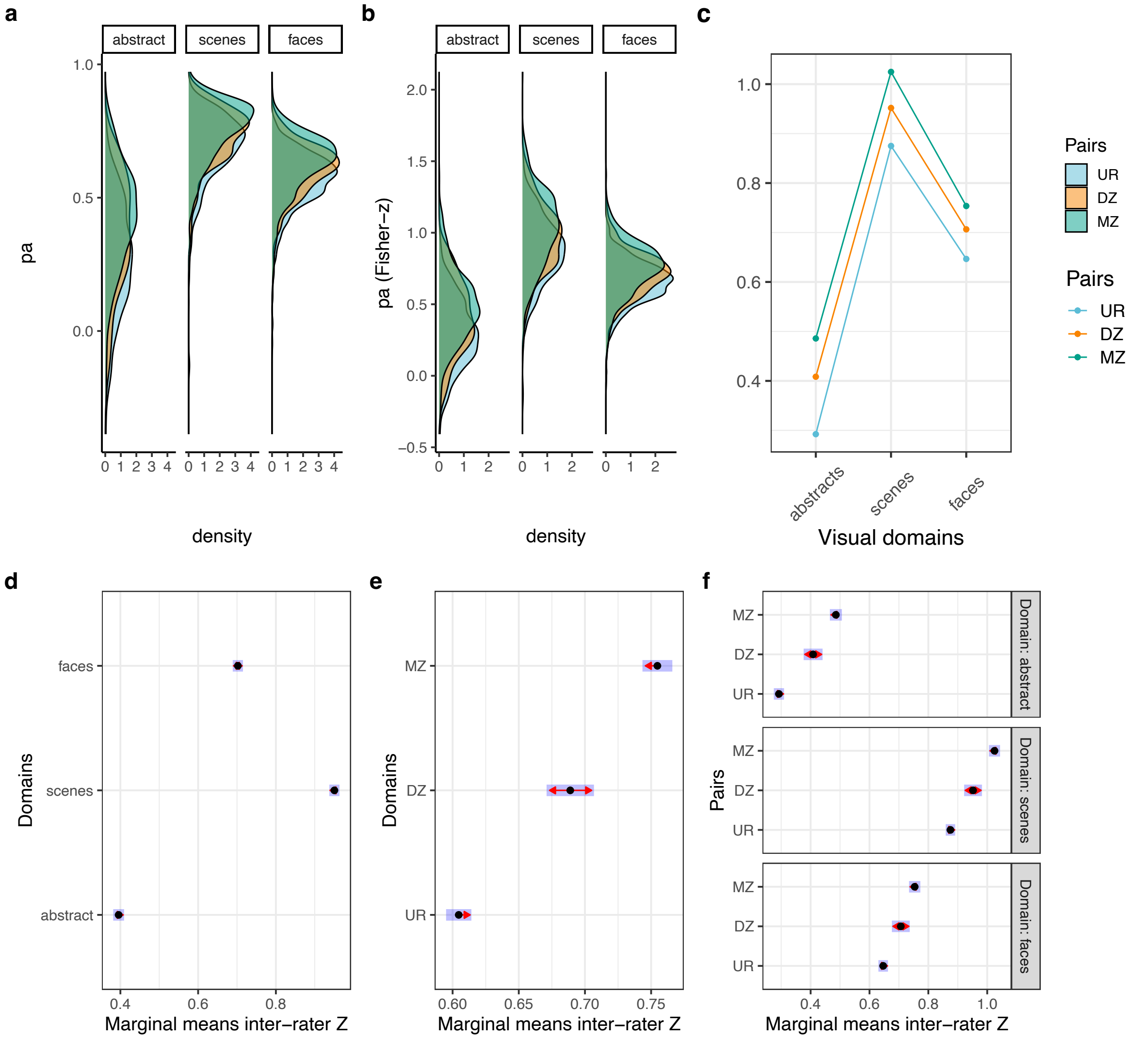

Supplementary Figure 6. *Pairwise agreement distribution and analysis in the main sample* [1]*.*

Panel **a** shows the density distribution of the pairwise agreement per different visual domains. The colours represent the pair class. Panel **b** shows the same distribution, but Fisher-*z* transformed. Panel **c** shows the average of the Fisher-z pairwise agreement scores across different visual domains and pair classes. In panels **d** to **f** the standard outputs from the emmeans R package show the marginal mean scores per comparison with relative 95% Confidence Intervals (CI).

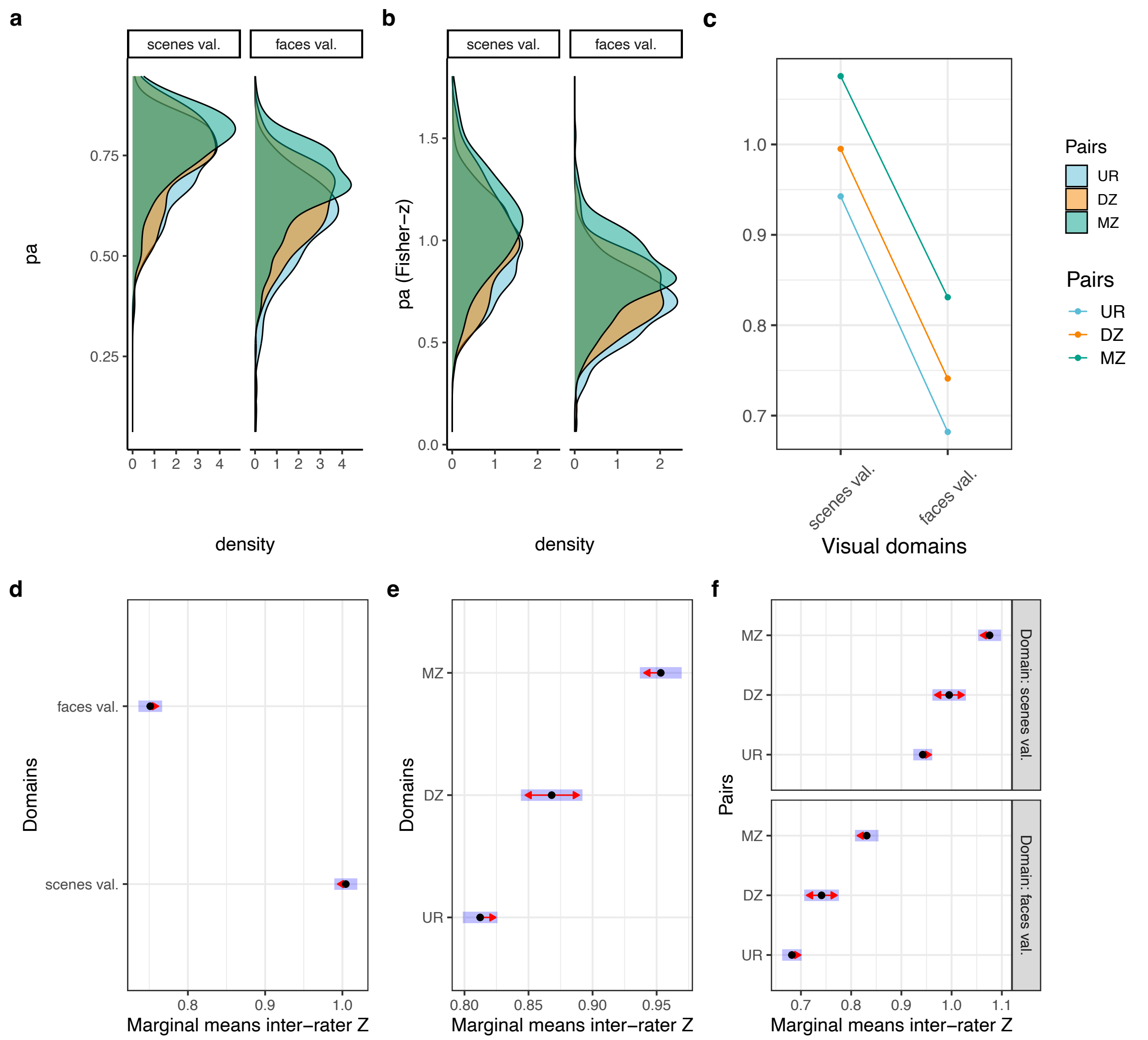

Supplementary Figure 7**.** Pairwise agreement distribution and analysis in the validation sample [2]**.**

Panel **a** shows the density distribution of the pairwise agreement per different visual domains. The colours represent the pair class. Panel **b** shows the same distribution, but Fisher-z transformed. Panel **c** shows the average of the fisher-z pairwise agreement scores across different visual domains and pair classes. In panels **d** to **f** the standard outputs from the emmeans R package show the marginal mean scores per comparison with relative 95% Confidence Intervals (CI).

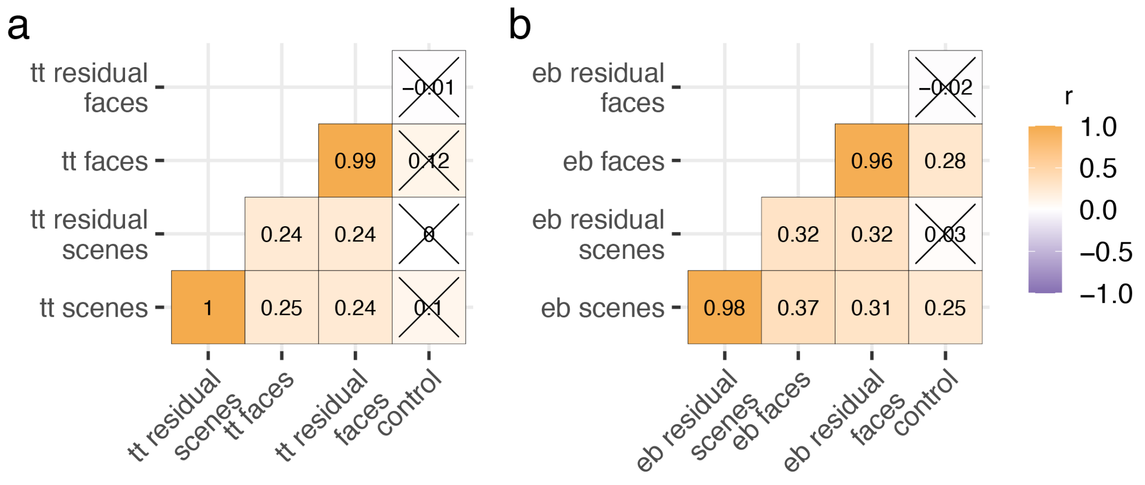

Supplementary Figure 8**.** *Phenotypic correlations persist after sensitivity analysis.*
a shows the phenotypic correlation matrices between the taste-typicality (tt) scores before and after regressing the control scores. **b** shows the phenotypic correlation matrices between the evaluation-bias (eb) scores before and after regressing the control scores. tt = Taste-typicality; eb = Evaluation-bias.

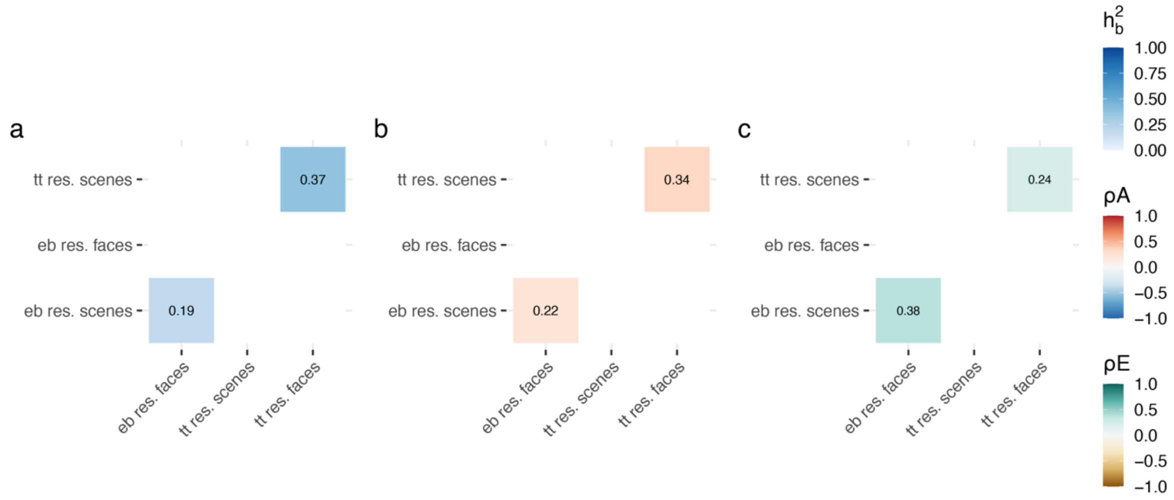

Supplementary Figure 9. *Bivariate heritability and genetic and environmental correlations persist after sensitivity analysis.*
Panel **a** shows the bivariate heritability (h^2_b) for taste-typicality (tt) and evaluation-bias (eb) after regressing the control scores. Panel **b** shows the genetic correlations (*ρA*) for taste-typicality (tt) and evaluation-bias (eb) after regressing the control scores. Panel **c** shows the environmental correlations (*ρE*) for taste-typicality (tt) and evaluation-bias (eb) after regressing the control scores. Estimates were obtained via a direct symmetric approach. [9]. tt = Taste-typicality; eb = Evaluation-bias; res. = residualised.

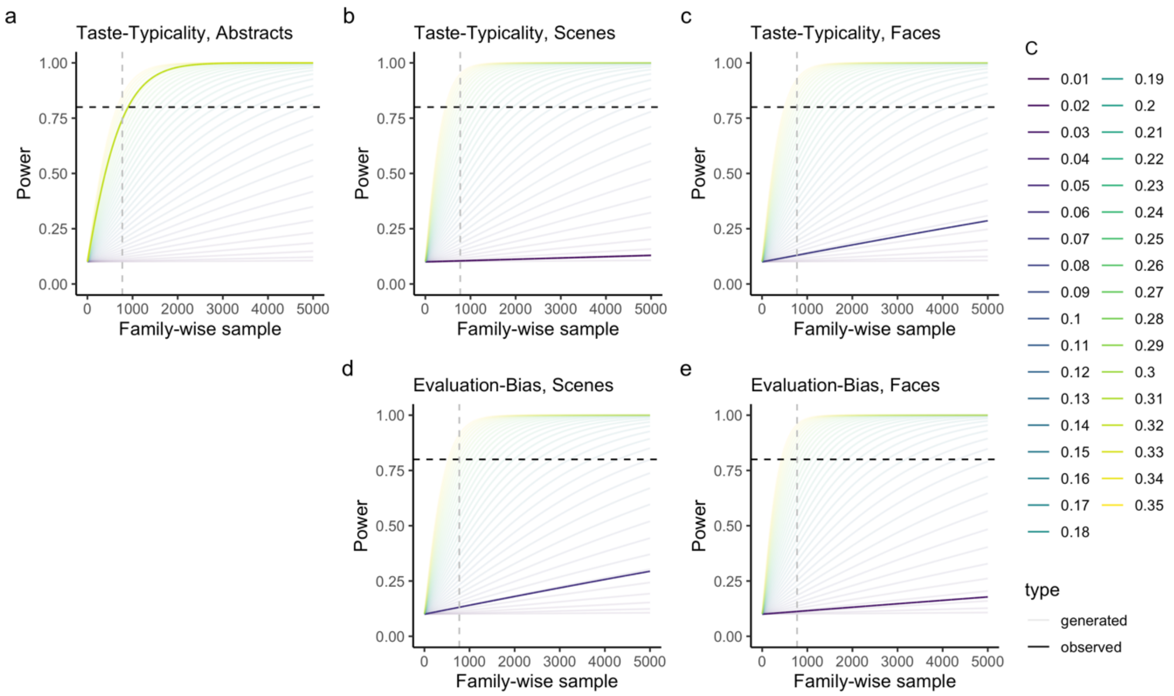

Supplementary Figure 10. *Power analysis.*

Power curves to detect C, given a fixed h^2 and a fixed MZ and DZ twin ratio, as a function of the total number of families. Colours represent different values of C. The horizontal dashed line represents a statistical power of 80%, while the vertical dashed line represents the number of families in the discovery sample. The shading represents the type of C: generated (low shading) *vs* observed (high shading) values for C.

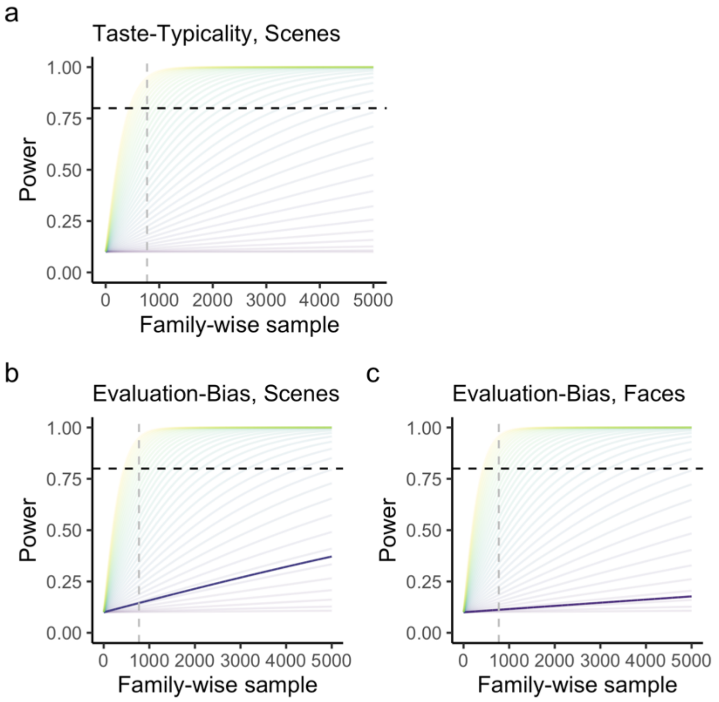

Supplementary Figure 11. *Power analysis in the validation sample.*
Power curves to detect C, given a fixed h^2 and a fixed MZ and DZ twin ratio, as a function of the total number of families in the validation sample. Colours represent different values of C. The horizontal dashed line represents a statistical power of 80%, while the vertical dashed line represents the number of families in the discovery sample. The shading represents the type of C: generated (low shading) *vs* observed (high shading) values for C.

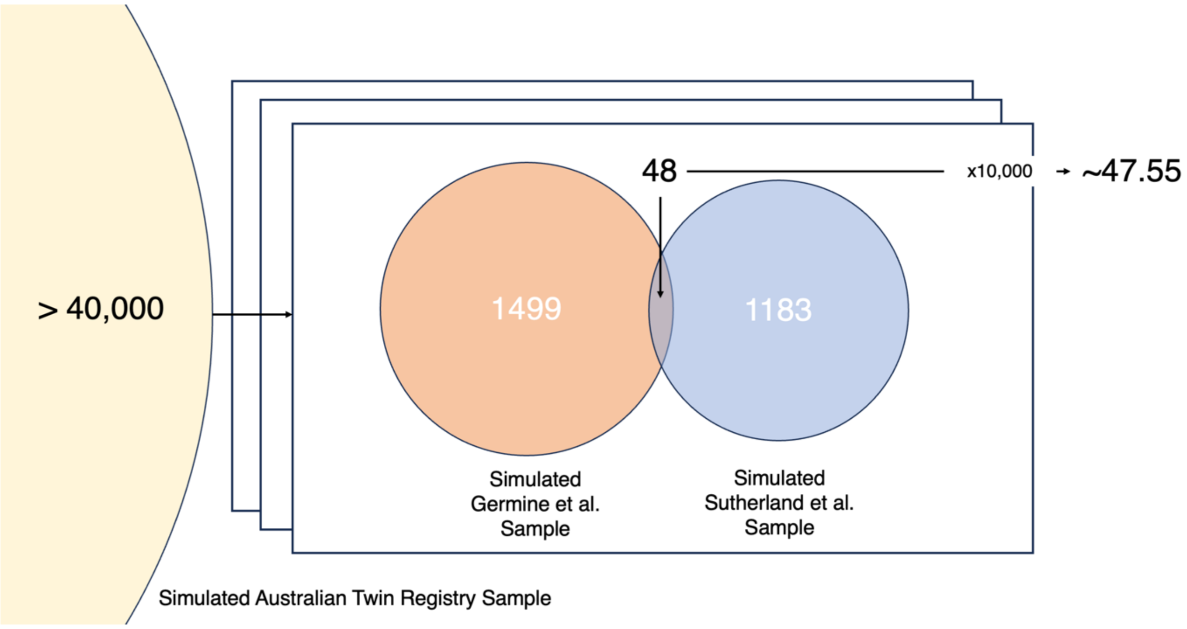

Supplementary Figure 12. Sample overlap.
Simulated average sample overlap between two simulated samples of equal size to the Germine et al. and Sutherland et al. study, drawn from a simulated registry with 40,000 individuals. On the right, the approximated average of the sample overlaps across 10,000 iterations.

Supplementary Tables

Supplementary Table 1: *Phenotypic Twin correlations before and after controlling for confounding effects*

phenotype	Images	MZ r	MZ 95% CI	DZ r	DZ 95% CI
Taste typicality					
	Abstract images	.29	[.20; .37]	.30	[.17; .41]
	Scenes	.36	[.28; .43]	.19	[.05; .32]
	Scenes val.	.30	[.18; .40]	.15	[.01; .28]
	Faces	.30	[.23; .38]	.19	[.03; .33]
	Faces val.	.33	[.22; .43]	.08	[-0.09; .25]
Evaluation bias					
	Abstract images	.34	[.25; .41]	.09	[-0.05; .22]
	Scenes	.26	[.17; .33]	.16	[.02; .29]
	Scenes val.	.31	[.20; .40]	.19	[.02; .34]
	Faces	.40	[.33; .47]	.22	[.09; .34]
	Faces val.	.31	[.21; .41]	.18	[-0.04; .37]
Taste typicality residuals					
	Scenes	.29	[.17; .39]	.13	[-0.01; .27]
	Faces	.31	[.20; .41]	.05	[-0.12; .21]
Evaluation bias residuals					
	Scenes	.28	[.17; .37]	.13	[-0.04; .29]
	Faces	.29	[.18; .39]	.20	[-0.02; .38]

Note: MZ r = Correlation between monozygotic twins. DZ r = Correlation between dizygotic twins. CI = Confidence interval. Taste-typicality and evaluation-bias residuals show phenotypic correlations after accounting for overall typicality and bias scores.

Supplementary Table 2: ***Univariate modelling of genetic and environmental contributions to inter-individual differences in aesthetic evaluation after controlling for confounding effects***

Images	Model	-2LL, AIC	df,χ^2	A	C	E
Taste typicality residuals						
Scenes	AE	263.75, 273.75	1204, 0.01	0.28 [0.18; 0.38]	0	0.72 [0.62; 0.82]
Faces	AE	-455.28, -445.28	1161,1.45	0.29 [0.18; 0.39]	0	0.71 [0.61; 0.82]
Evaluation bias residuals						
Scenes	AE	2801.30, 2810.30	1203, 0.01	0.27 [0.17; 0.37]	0	0.73 [0.63; 0.83]
Faces	AE	3084.44, 3094.44	1165, 0.27	0.29 [0.19; 0.39]	0	0.71 [0.61; 0.81]

Note: -2LL (- 2 Log likelihood); AIC (Akaike Information Criteria); df (Degrees of freedom); A (Additive genetic); C (Common environmental); E (Unique environmental and error). Sex and age are included as covariates in every model. Taste-typicality and evaluation-bias residuals are the residuals of taste-typicality and evaluation-bias regressed on control-typicality and control-bias. Significance and comparative statistics for the reduced models (AE, CE, and E) are obtained by comparison with the respective full ACE model (all ACE models fit were judged to be good in comparison with the respective full saturated models).

Supplementary References

1. Germine, L. *et al.* Individual Aesthetic Preferences for Faces Are Shaped Mostly by Environments, Not Genes. *Curr. Biol.* **25**, 2684–2689 (2015).
2. Sutherland, C. A. M. *et al.* Individual differences in trust evaluations are shaped mostly by environments, not genes. *Proc. Natl. Acad. Sci.* **117**, 10218–10224 (2020).
3. Vessel, E. A., Maurer, N., Denker, A. H. & Starr, G. G. Stronger shared taste for natural aesthetic domains than for artifacts of human culture. *Cognition* **179**, 121–131 (2018).
4. Chen, Y.-C. *et al.* “Taste typicality” is a foundational and multi-modal dimension of ordinary aesthetic experience. *Curr. Biol.* S0960982222002615 (2022) doi:10.1016/j.cub.2022.02.039.
5. Kurosu, A. & Todorov, A. The shape of novel objects contributes to shared impressions. *J. Vis.* **17**, (2017).
6. Vessel, E. A. & Rubin, N. Beauty and the beholder: Highly individual taste for abstract, but not real-world images. *J. Vis.* **10**, 18–18 (2010).
7. Zeki, S. & Chén, O. Y. The Bayesian-Laplacian brain. *Eur. J. Neurosci.* **51**, 1441–1462 (2020).
8. Hönekopp, J. Once more: Is beauty in the eye of the beholder? Relative contributions of private and shared taste to judgments of facial attractiveness. *J. Exp. Psychol. Hum. Percept. Perform.* **32**, 199–209 (2006).
9. Verhulst, B., Prom-Wormley, E., Keller, M., Medland, S. & Neale, M. C. Type I Error Rates and Parameter Bias in Multivariate Behavioral Genetic Models. *Behav. Genet.* **49**, 99–111 (2019).
10. Verhulst, B. A Power Calculator for the Classical Twin Design. *Behav. Genet.* **47**, 255–261 (2017).
11. Hopper, J. L. The Australian Twin Registry. *Twin Res. Off. J. Int. Soc. Twin Stud.* **5**, 329–336 (2002).
12. Hopper, J. L., Foley, D. L., White, P. A. & Pollaers, V. Australian Twin Registry: 30 years of progress. *Twin Res. Hum. Genet. Off. J. Int. Soc. Twin Stud.* **16**, 34–42 (2013).
