# Supplementary material for: Genetic effects on variability in visual aesthetic evaluations are partially shared across visual domains
Source: Commun Biol. 2024 Jan 6;7:55. doi: 10.1038/s42003-023-05710-4 (PMC10771521; doi:10.1038/s42003-023-05710-4)
Supplement: Supplementary file 3 — Description of Additional Supplementary Files [file 42003_2023_5710_MOESM3_ESM.pdf]

Description of Additional Supplementary Files

File name: Supplementary Data 1
Description: Assumptions test results for twin’s means and variances

File name: Supplementary Data 2
Description: Full ACDE estimates

File name: Supplementary Data 3
Description: ACDE 95% confidence intervals

File name: Supplementary Data 4
Description: The source data behind Figure 1b

File name: Supplementary Data 5
Description: The source data behind individual-level data points in Figure 2a

File name: Supplementary Data 6
Description: The source data behind group-level data point in Figure 2b

File name: Supplementary Data 7
Description: The source data behind individual-level data points in Figure 2c

File name: Supplementary Data 8
Description: The source data behind group-level data points in Figure 2d
