# Supplementary material for: Genetic effects on variability in visual aesthetic evaluations are partially shared across visual domains
Source: Commun Biol. 2024 Jan 6;7:55. doi: 10.1038/s42003-023-05710-4 (PMC10771521; doi:10.1038/s42003-023-05710-4)
Supplement: Supplementary file 12 — Reporting Summary [file 42003_2023_5710_MOESM12_ESM.pdf]

nature portfolio

Corresponding author(s): Giacomo Bignardi

Last updated by author(s): Dec 5, 2023

Reporting Summary

Nature Portfolio wishes to improve the reproducibility of the work that we publish. This form provides structure for consistency and transparency in reporting. For further information on Nature Portfolio policies, see our Editorial Policies and the Editorial Policy Checklist.

Statistics

For all statistical analyses, confirm that the following items are present in the figure legend, table legend, main text, or Methods section.

n/a	Confirmed	
☐	☒	The exact sample size (*n*) for each experimental group/condition, given as a discrete number and unit of measurement
☐	☒	A statement on whether measurements were taken from distinct samples or whether the same sample was measured repeatedly
☐	☒	The statistical test(s) used AND whether they are one- or two-sided<br>*Only common tests should be described solely by name; describe more complex techniques in the Methods section.*
☐	☒	A description of all covariates tested
☐	☒	A description of any assumptions or corrections, such as tests of normality and adjustment for multiple comparisons
☐	☒	A full description of the statistical parameters including central tendency (e.g. means) or other basic estimates (e.g. regression coefficient) AND variation (e.g. standard deviation) or associated estimates of uncertainty (e.g. confidence intervals)
☐	☒	For null hypothesis testing, the test statistic (e.g. *F*, *t*, *r*) with confidence intervals, effect sizes, degrees of freedom and *P* value noted<br>*Give P values as exact values whenever suitable.*
☒	☐	For Bayesian analysis, information on the choice of priors and Markov chain Monte Carlo settings
☐	☒	For hierarchical and complex designs, identification of the appropriate level for tests and full reporting of outcomes
☐	☒	Estimates of effect sizes (e.g. Cohen's *d*, Pearson's *r*), indicating how they were calculated

Our web collection on statistics for biologists contains articles on many of the points above.

Software and code

Policy information about availability of computer code

Data collection	No data was collected in this study.
Data analysis	R; R packages used in the analysis and rationale: readr (reading data into r); tidyverse (cleaning data and data work flow); tidylog (checking step by step data cleaning); Rstatix (descriptive statistics); lme4 (multilevel modeling and variance component analysis); pbkrtest (confidence intervals of for the variance partition component); psych (Fisher Z transformations); emmeans (Pairwise comparisions); effectsize (compute effect sizes); umx (residualization of covariates); OpenMx (Structural Equation Modeling); ggplot2 (Plotting); ggstatsplot (Plotting); patchwork (Plotting).

For manuscripts utilizing custom algorithms or software that are central to the research but not yet described in published literature, software must be made available to editors and reviewers. We strongly encourage code deposition in a community repository (e.g. GitHub). See the Nature Portfolio guidelines for submitting code & software for further information.

Data

Policy information about availability of data

All manuscripts must include a data availability statement. This statement should provide the following information, where applicable:

- Accession codes, unique identifiers, or web links for publicly available datasets
- A description of any restrictions on data availability
- For clinical datasets or third party data, please ensure that the statement adheres to our policy

All data were made available by the original authors of the first two studies, Germine et al. and Sutherland et al., and can be found at https://osf.io/c3hz6/ and https://osf.io/35zf8/?view_only=e76c6755dcea4be2adc5b075cae896e8, respectively.

Research involving human participants, their data, or biological material

Policy information about studies with human participants or human data. See also policy information about sex, gender (identity/presentation), and sexual orientation and race, ethnicity and racism.

Reporting on sex and gender	Findings apply to participants sex only. As far as the authors of this analysis are aware of, gender was not considered in the previous data collection.
Reporting on race, ethnicity, or other socially relevant groupings	We did not consider additional groupings in our analysis.
Population characteristics	Twin sample from Australian population with a mean age 45 y (sd = 13 y, ranging from 21 to 68 y) for the Germine et al. sample and 47 y (sd = 15 y, ranging from 16 to 80 y), for the Sutherland et al., sample, respectively.
Recruitment	This study did not involved recruitment of participants. Details on recruitment can be found in Germine et al. and Sutherland et al., at https://osf.io/c3hz6/ and https://osf.io/35zf8/?view_only=e76c6755dcea4be2adc5b075cae896e8, respectively.
Ethics oversight	The original studies were approved by the Committee for the Use of Human Subjects at Harvard University, University of Western Australia, and at Twins Research Australia. Participants gave informed consent before taking part in the original studies.

Note that full information on the approval of the study protocol must also be provided in the manuscript.

Field-specific reporting

Please select the one below that is the best fit for your research. If you are not sure, read the appropriate sections before making your selection.

☐ Life sciences ☒ Behavioural & social sciences ☐ Ecological, evolutionary & environmental sciences

For a reference copy of the document with all sections, see nature.com/documents/nr-reporting-summary-flat.pdf

Behavioural & social sciences study design

All studies must disclose on these points even when the disclosure is negative.

Study description	Quantitative approach; Classical Twin Design.
Research sample	Same-sex twin pairs originally recruited by the Twin Research Australia. Details are given in Germine et al., and Sutherland et al.
Sampling strategy	Sampling strategy was defined in function of data availability and previous data collection.
Data collection	No data were collected in the study. Data collection details are given in Germine et al., and Sutherland et al.
Timing	N.A.
Data exclusions	Following Vessel et al., and Chen et al., we excluded individuals with low intra-rater rating reliability. We excluded pairs and individuals with metrics with values above three times the Inter Quartile Range (IQR). For the latter exclusion, we reported results for both analyses carried out on the sample excluding and including participants.
Non-participation	N.A.
Randomization	Participants were not allocated to random groups. Age and sex were regressed out from the main variables of interests in the main analysis.

Reporting for specific materials, systems and methods

We require information from authors about some types of materials, experimental systems and methods used in many studies. Here, indicate whether each material, system or method listed is relevant to your study. If you are not sure if a list item applies to your research, read the appropriate section before selecting a response.

Materials & experimental systems

n/a	Involved in the study
☒	☐ Antibodies
☒	☐ Eukaryotic cell lines
☒	☐ Palaeontology and archaeology
☒	☐ Animals and other organisms
☒	☐ Clinical data
☒	☐ Dual use research of concern
☒	☐ Plants

Methods

n/a	Involved in the study
☒	☐ ChIP-seq
☒	☐ Flow cytometry
☒	☐ MRI-based neuroimaging

Plants

Seed stocks	*Report on the source of all seed stocks or other plant material used. If applicable, state the seed stock centre and catalogue number. If plant specimens were collected from the field, describe the collection location, date and sampling procedures.*
Novel plant genotypes	*Describe the methods by which all novel plant genotypes were produced. This includes those generated by transgenic approaches, gene editing, chemical/radiation-based mutagenesis and hybridization. For transgenic lines, describe the transformation method, the number of independent lines analyzed and the generation upon which experiments were performed. For gene-edited lines, describe the editor used, the endogenous sequence targeted for editing, the targeting guide RNA sequence (if applicable) and how the editor was applied.*
Authentication	*Describe any authentication procedures for each seed stock used or novel genotype generated. Describe any experiments used to assess the effect of a mutation and, where applicable, how potential secondary effects (e.g. second site T-DNA insertions, mosiacism, off-target gene editing) were examined.*
